# Sustainability assessment and improvement strategies research for typical arid and resource-developing regions

**DOI:** 10.1371/journal.pone.0251088

**Published:** 2021-05-04

**Authors:** Xudong Zhou

**Affiliations:** Luzhou Vocational and Technical College, Luzhou, Sichuan Province, China; Institute for Advanced Sustainability Studies, GERMANY

## Abstract

Located in the Eurasian continent’s hinterland, Xinjiang is a typical arid and resource-developing region in China’s northwest. Problems such as excessive resource consumption, environmental pollution, and ecological imbalance are becoming severe, which have become the bottleneck that further restricts Xinjiang’s sustainable development. Due to its outstanding quantitative advantages, ecological efficiency has become a significant indicator and analytical tool for measuring the green economy and sustainable development. In this study, we analyzed ecological efficiency variation for Xinjiang’s 14 prefectures between 2001 and 2015 using a super-efficient data envelopment model (DEA), Malmquist Index, and Tobit model. These analyses indicated that: (1) The overall ecological efficiency level of Xinjiang is low, and development among regions is unbalanced, out of sync, lacks sustainability. (2) From 2001 to 2015, Xinjiang’s ecological efficiency showed a W-shaped rising trend and finally increased by 5.7%. It is due to the substantial improvement in environmental efficiency. (3) By analyzing the environmental efficiency and resource efficiency, 14 prefectures in Xinjiang consist of four development modes: low energy consumption and low emission, high energy consumption and low emission, low energy consumption and high emission, and high energy consumption and high emission. (4) Water resources are restricting factors of arid regions. In most prefectures, there exist excessive water resource investment, excessive COD, and NH_3_-N emissions. (5) By analyzing the Malmquist index, it shows that the technical progress index(TC) restricted ecological efficiency. In contrast, the technical efficiency index (EC) promoted ecological efficiency.(6)The ecological efficiency was positively correlated with the utilization of foreign capital, urbanization rate, and average education degree but negatively correlated with the marketization degree. The study has guidance and reference function for the sustainable development of Xinjiang—a vital corridor of the Silk Road Economic Belt, and also provides a reference to the research work of other arid resource-based regions.

## Introduction

In 1990, some scholars first proposed the ecological efficiency concept, which meant the added value ratio to the added environmental impact [1]. Later, other scholars defined the concept of ecological efficiency. "The ecological efficiency must provide a product or service which has a price competition advantage to meet human needs and ensure the quality of life and can reduce the ecological impact resources consumption intensity of the products or services, and the degree of reduction should be identical with the estimation of the earth carrying capacity" [2]. Ecological efficiency has received significant attention on sustainable development [3] and has become a hot spot for researchers and industrial ecology [4, 5] and become a useful analytical tool to measure sustainable development.

The model method is used in ecological efficiency research, among which the data envelopment method (DEA) is widely used. Western scholars have made earlier studies on the data envelopment model. At the beginning of this century, Dychkhoff [6], Sarkis [7], Korhonen [8], and Kuosmanen [9] have studied DEA models and applied them to power plant transportation, and transportation. Chinese scholars have also applied the DEA method of the ecological efficiency evaluation of provinces [10, 11] and regions [12, 13]. In various attempts to optimize the traditional DEA, the super-efficiency DEA model has been widely used. Shanshan Li [14] measured and analyzed the ecological efficiency of 30 provinces and prefectures in China. Lina Fu et al. [15] measured and analyzed the ecological efficiency of the "3+5" city clustered in the Changsha-Zhuzhou-Xiangtan area. Zhimin Dai et al. [16] measured and analyzed the industrial ecological efficiency of several provinces in East China. Apply research on ecological efficiency focuses on the three levels of enterprise [17–19], industry [20–22], and region [23–25]. Some scholars have studied the temporal and spatial distribution, dynamic changes, and influencing factors of ecological efficiency in provinces such as Beijing [26], Shenzhen [27], Jilin [28], Jiangsu [29], and Jiangxi [30].

**Fig 1:**
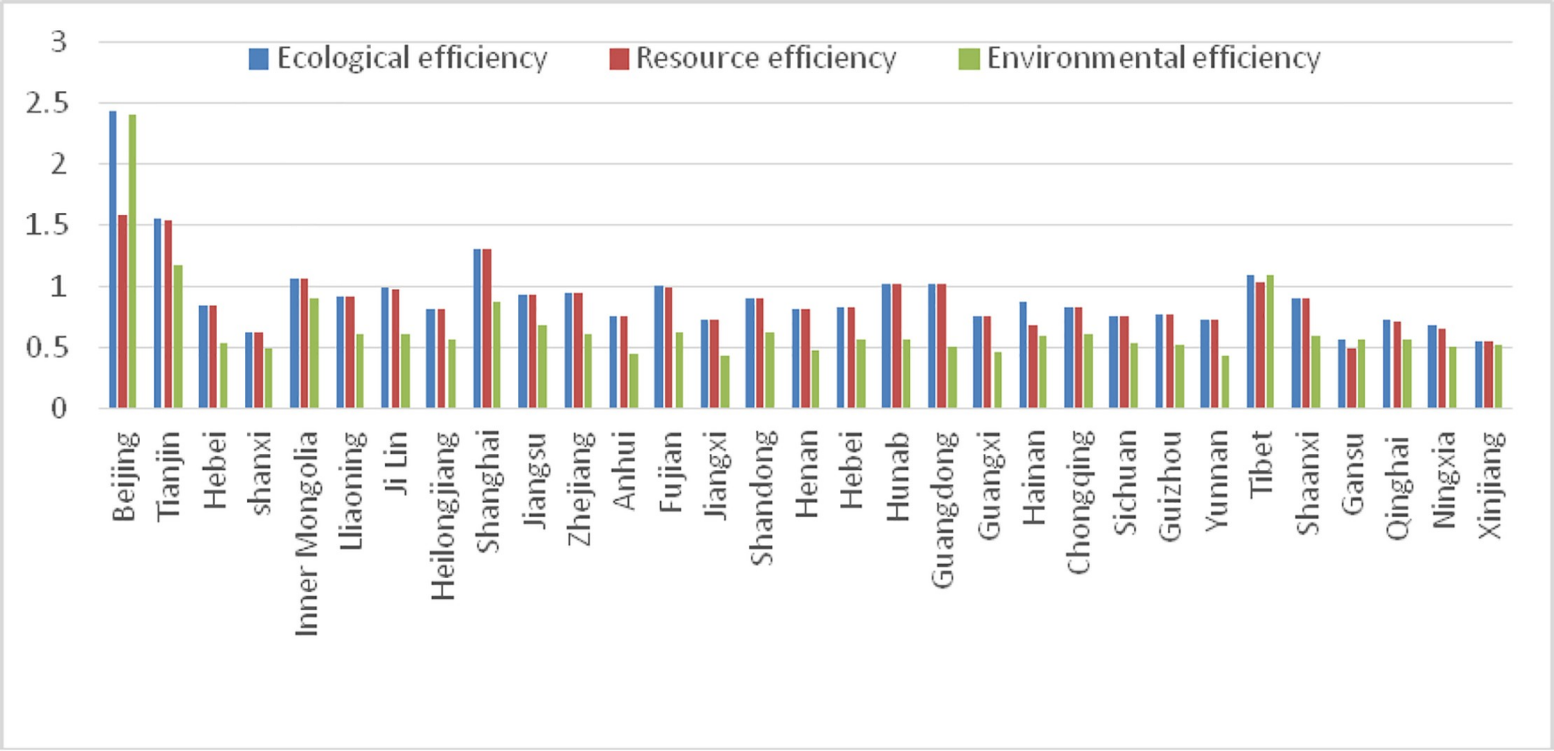
Comparison of ecological efficiency among different provinces in China.

Xinjiang lies on China’s western border and the Eurasian continent hinterland (Fig 1), with desert area accounting for 60% of China’s desert area. It is one of the world’s drought centers of a fragile ecological environment. Xinjiang is a significant corridor of the Silk Road Economic Belt. Meanwhile, coal, oil, natural gas, and other mineral resources are abundant [31], and the Chinese Government [32] list about 60% of prefectures in Xinjiang as resource-based prefectures and regions. Problems such as excessive resource consumption, environmental pollution, and ecological imbalance are becoming increasingly severe, which have become the bottleneck that further restricts Xinjiang’s sustainable development. Thus, it is crucial to measure the ecological level and efficiency of economic development in this region, which is also the focus of policymakers at all levels. There are few and incomplete research data on ecological efficiency in Xinjiang [33–35]. The article uses the method of super-efficiency DEA model, Malmquist Index, and Tobit model. It utilizes 15 consecutive years (from 2001 to 2015) of data onto14 prefectures of Xinjiang panel data as samples. The present study’s goal was to explore annual changed trends in ecological efficiency, spatial distribution patterns, analyze the existing shortcomings, explore the ways of improvement and promotion, and provide a reference to the sustainable development of resource-developing prefectures in arid areas.

## Materials and methods

### Research methods

#### Super-efficiency DEA model

The DEA-CCR model was used to assess decision units’ effectiveness using the "multi-input and multi-output" model [36]. In 1993, Andersen et al. [37] proposed an improved CCR model- the super-efficiency DEA model. It overcame the CCR model’s defect, which could not make further evaluation and comparison of multiple decision- making units (DMU) and enabled effective decision-making units to make comparison and ordering.

The super-efficient DEA model is given as:

$$\begin{array}{l} \;\mathrm{Min}\theta \\ s.t.\{\begin{array}{l} \sum_{i=1,j\neq 1}^{n}X_{j}\lambda_{j}+s^{-}=\theta X_{0}\\ \sum_{i=1,j\neq 1}^{n}X_{j}\lambda_{j}-s^{+}=Y_{0}\\ \lambda_{j}\geq 0,\;J=1,\;2,\ldots k-1,\;k\\ s^{-}\geq 0,\;s^{+}\geq 0 \end{array} \end{array}$$ (1)

In formula (1), θ represents the decision unit’s efficiency value; X and Y represent input variables and output variables, respectively, and λ represents the combined ratio in a practical decision-making unit used to judge the scale of the benefit of DMU. ∑λ<1, ∑λ = 1, and ∑λ>1 denotes an increase, invariance, and decreases in scale benefits, respectively. S- and S+ represent relaxation and residual variables, respectively. θ < 1 and S- ≠0 or S+ ≠0 indicate that the decision unit does not reach the optimal efficiency, whereas θ≥1 and S- ≥0 and S+≥0 indicate that the decision unit achieves optimal efficiency.

#### The Malmquist index

The Swedish economist Malmquist [38] first proposed the Malmquist index in 1953. Caves [39] proposed that the Malmquist index represented the total factor production efficiency under multi-input and multi-output conditions and integrated it into the DEA model to calculate the production’s total factor productivity sector. The basic principles of this implementation are as following:

The Malmquist index from time interval t to t+1 can be expressed as [40]:

$$\begin{array}{l} \mathrm{TFP}={\left[\frac{D^{t}\left(x_{t+1}•y_{t+1}\right)}{D^{t}\left(x_{t}•y_{t}\right)}\times \frac{D^{t+1}\left(X_{t+1}•y_{t+1}\right)}{D^{t+1}\left(x_{t}•y_{t}\right)}\right]}^{\frac{1}{2}}\\ \;={\left[\frac{D^{t}\left(x_{t+1}•y_{t+1}\right)}{D^{t+1}\left(x_{t+1}•y_{t+1}\right)}\times \frac{D^{t}\left(x_{t}•y_{t}\right)}{D^{t+1}\left(x_{t}•y_{t}\right)}\right]}^{\frac{1}{2}}\times \frac{D^{t+1}\left(x_{t+1}•y_{t+1}\right)}{D^{t}\left(x_{t}•y_{t}\right)}\\ \;=\mathrm{TC}\times \mathrm{EC}\\ \;=\mathrm{TC}\times \mathrm{PE}\times \mathrm{SE} \end{array}$$ (2)

In formula (2), the Malmquist index variation is the total factor productivity (TFP) variation and represents the change in the degree of productivity for a decision unit from t to t+1. TFP > 1 indicates an increase in productivity, whereas TFP < 1 indicates a decrease in productivity. The TFP can be subdivided into technical change (TC) and efficiency change (EC).TC refers to moving the production frontier to productivity, while EC contributes from changing technical efficiency to productivity between the period encompassed by t and t+1. EC can be further divided into PE (pure technical efficiency) and SE (scale efficiency).

#### Tobit model

The ecological efficiency estimated by the DEA model is not just affected by the input and output indicators that are selected but also by other factors. Collet [41] developed a two-step method based on the DEA to identify the factors influencing ecological efficiency. The first step is to evaluate the efficiency of decision-making units by the DEA. A regression model is then established that uses the estimated efficiency value as the dependent variable and influential factors as the independent variables. Independent variables’ coefficients then determine the orientation and intensity of the influential factors on environmental efficiency.

The Tobit model can be written as:

$$y_{i}=\{\begin{array}{l} y_{i}^{*}=x_{i}\beta +\varepsilon_{i}\;y_{i}^{*}\geq 0\\ 0\;y_{i}^{*}\leq 0 \end{array}$$ (3)

In formula (3), x_i_ is the independent variable, y_i_ is the observed dependent variable, y_i_* is the latent variable, β is the correlation coefficient, ε_i_ is the independent variable. The disturbance term is ε_i_-N (0, σ^2^).

### Selection of evaluation indicators and data source

The fundamental concept of increasing ecological efficiency goes to maximize its value with minimal resource consumption and environmental pollution. These objectives are consistent with the requirements for the input and output of the DEA method. In practice, profitability is described as the output index, while cost is used as the input index.

This paper considered environmental, economic and resource factors here concerning previous studies [42–44]. We selected several parameters as input indices, including energy consumption, power consumption, water consumption, and fixed assets investment, the employment of five resource consumption indices, exhaust emissions, waste-water discharge, and solid waste discharge from three kinds of environmental pollution parameters. Besides, we considered the economic value as the output index (See Table 1). We constructed an ecological efficiency evaluation system using these indices. This paper acquired the input and output data used in this study from the Statistical Yearbooks of Xinjiang Uygur Autonomous Region, the yearbooks of Xinjiang Environmental Statistics, and statistical yearbooks of the prefectures.

**Table 1:** Evaluation indicators and statistical data used in eco-efficiency analysis.

Evaluation indicators	Average	SD	Max	Min
Input				
Total energy consumed(10^4^ t)	8196.68	3954.29	15651.2	3496.44
Total electricity consumption(10^8^kwh)	751.62	637.41	2190.68	184.62
Total water(10^8^m^3^)	523.95	33.53	588.05	475
Fixed investments(10^8^ yuan RMB)	3754.16	3240.86	10729.32	706.00
Employed population(10^4^people)	885.70	155.55	1195.06	685.38
SO_2_ emissions(10^4^t)	53.76	12.97	68.39	29.61
NO_x_ emissions(10^4^t)	48.27	17.60	75.42	21.34
NH_3_-N emissions(10^4^t)	2.82	0.94	4.19	1.79
COD emissions(10^4^t)	35.96	15.19	57.94	20.1
General industrial solid waste(10^4^t)	3730.07	2972.56	9283.05	783.6
Output				
GDP(10^8^ yuan RMB)	4756.25	2715.70	9324.8	1491.6

## Results and analysis

### Static ecological efficiency measurement and analysis

The ecological efficiency index is a comprehensive index considering the environment and resources. This paper decomposed ecological efficiency into environmental efficiency and resource efficiency for measurement and analysis and analyzed the ecological efficiency in Xinjiang through the changing trend of the three. Depending on relevant studies [45, 46], environmental efficiency can be expressed by the ratio of economic output index to environmental emission index, while resource efficiency can be expressed by the ratio of economic output index to resource input index. This paper used Dea-solver PRO software and input-oriented super-efficiency DEA models to study Xinjiang’s ecological efficiency level from 2001 to 2015 and calculated the ecological efficiency, resource efficiency, and environmental efficiency from 2001 to 2015. Besides, this paper analyzed temporal and spatial changes from the provincial, area, and city levels.

#### Provincial-level measurement and analysis

**Fig 2:**
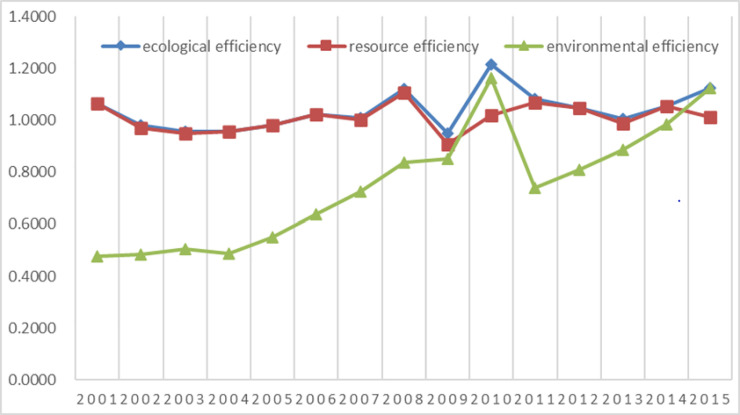
Ecological Efficiency of Xinjiang between 2001 and 2015.

**Fig 3:**
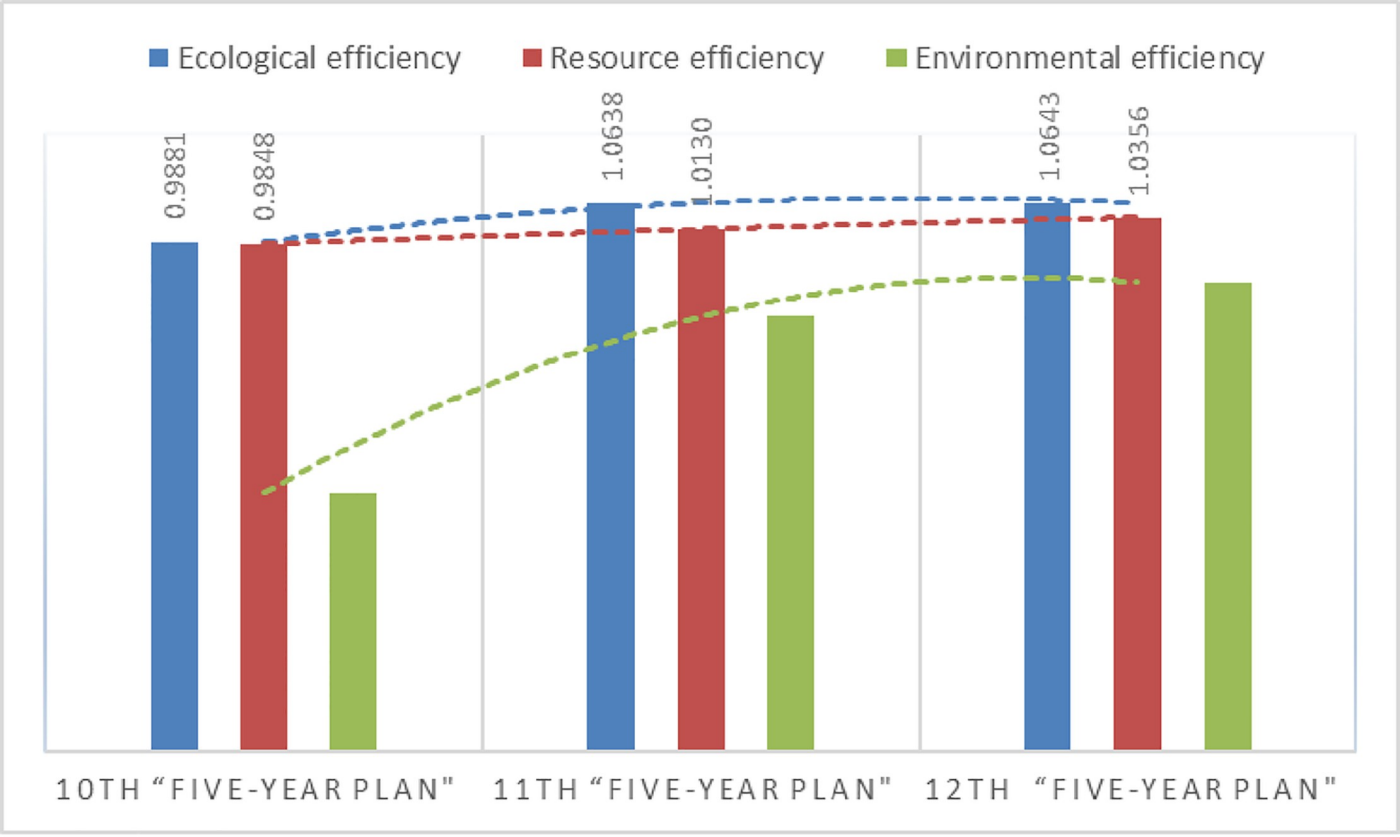
Variation of eco-efficiency during the “Tenth Five-Year Plan” to the “Twelfth Five-Year Plan”.

As shown in Figs 1–3 that:

(1) Xinjiang ranks 31st in China in terms of ecological efficiency, which is only 0.56, twice lower than the average value and 4.32 times different from the first place Beijing, indicating that the ecological efficiency level of Xinjiang is still very low. From the perspective of resource efficiency, it ranks 30th in China, which is still very low. However, Xinjiang has improved the ranking of environmental efficiency, ranking 22nd in China and up to nine places. It shows that dwindling ecological efficiency is due to low resource efficiency in Xinjiang; unreasonable resource allocation, large resource consumption, and the high energy consumption is still the characteristics and current economic development situation in Xinjiang. Compared with resource efficiency, environmental efficiency is low, but it has been improved. The "Five-in-One new pattern" and “ecological civilization thought” proposed at the 18th CPC National Congress has promoted environmental protection, and the emission of environmental pollutants has dropped. In 2015, Xinjiang ranked seventh from the bottom in China regarding the discharge of waste-water, which was lower than most provinces, indicating that Xinjiang’s high pollution phenomenon has been reduced.

(2) From 2001 to 2015, the ecological efficiency of Xinjiang was fluctuating. It first decreased from 2001 to 2005 and then increased from 2006 to 2008, it declined sharply in 2009; then rebounded to the peak in 2010; since then it has been kept in the efficient production frontiers, finally showed a small increase of 5.7%. Depending on the change analysis of the three five-year plans, the ecological efficiency increased from 0.9881 in the Tenth Five-Year Plan period to 1.0638 in the Eleventh Five-Year Plan period and 1.0643 in the Twelfth Five-Year Plan Period, showing a steady rise. From the beginning of the Eleventh Five-Year Plan period in 2006 to the end of the Twelfth Five-Year Plan period in 2015, except for a slight decline in 2009, the ecological efficiency has been stable at more than 1.00 and remained at the efficient production frontiers. It shows that since 2001, through the implementation of a series of policies and measures and the three “five-year plans”, Xinjiang’s ecological efficiency has been improved and enhanced in stages.

(3) To further investigate ecological efficiency variation, this paper decomposed it into resource efficiency and environmental efficiency. The variation trend of resource efficiency from 2001 to 2015 was consistent with ecological efficiency, which showed a continuous fluctuation, from the Tenth Five-Year Plan period to the Twelfth Five-Year Plan period showed a small rise. Environmental efficiency showed a sizable rising trend with the change of time. From 2001 at the beginning of the Tenth Five-Year Plan period to 2015 at the end of the Twelfth Five-Year Plan period, increasing by 2.35 times, growth of 135.7%, and realized a leap from low environmental efficiency to high environmental efficiency. These results indicate that Xinjiang achieved remarkable progress in environmental governance between the Tenth and Twelfth "Five-Year Plan" through the continuous prevention and control of environmental pollution.

#### Area level measurement and analysis

**Fig 4:**
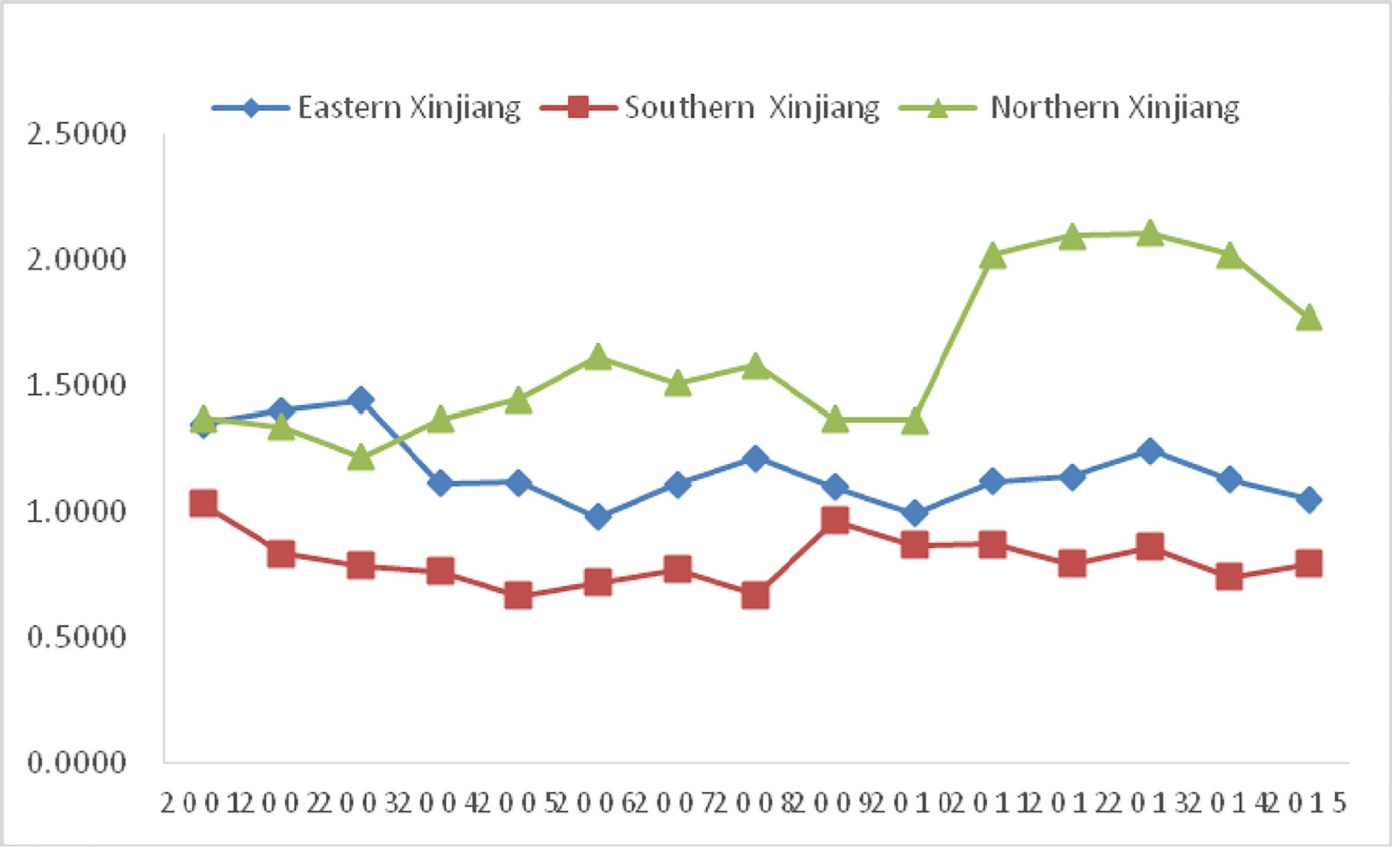
Variation trend of ecological efficiency in different regions of Xinjiang from 2001.

**Fig 5:**
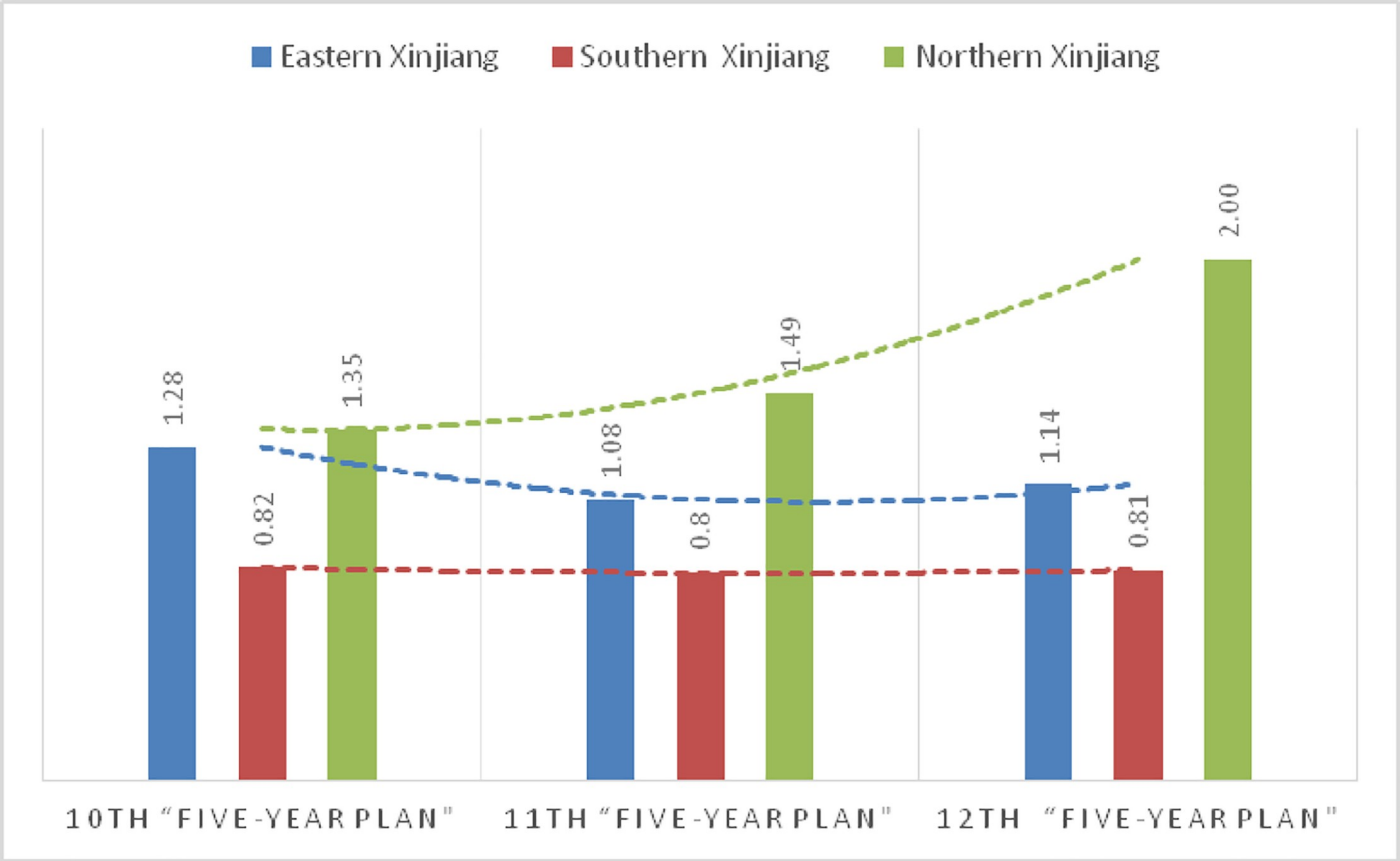
Variation trend of eco-efficiency during the “Tenth Five-Year Plan” to the “Twelfth Five-Year Plan”.

As shown in Table 2, Figs 4 and 5:

(1) Comparing the eco-efficiency values of Northern Xinjiang, Eastern Xinjiang, and Southern Xinjiang, they are 1.61, 1.16, and 0.81, respectively. The eco-efficiency of Northern Xinjiang is larger than that of Eastern Xinjiang and Southern Xinjiang. The eco-efficiency is deconstructed into the resource efficiency and the environmental efficiency for analysis, and the resource efficiency in Northern Xinjiang is higher than that of Southern Xinjiang and Eastern Xinjiang. The environmental efficiency in Northern Xinjiang is higher than that of Eastern Xinjiang, and that of Southern Xinjiang is the lowest among them. In general, the ecological efficiency in Southern Xinjiang is low, with ecological efficiency, resource efficiency, and environmental efficiency all less than 1.00.

(2) From 2001 to 2015, the ecological efficiency in Northern Xinjiang fluctuated and increased by 28.9%. Southern Xinjiang and Eastern Xinjiang showed a fluctuation decline. During the period from the Tenth Five-Year Plan period to the Eleventh Five-Year Plan period and then to the Twelfth Five-Year Plan period, the ecological efficiency of Northern Xinjiang and Eastern Xinjiang reached the efficient production frontier, while the ecological efficiency of Southern Xinjiang was low and did not reach the efficient production frontier. The ecological efficiency in Northern Xinjiang increased from 1.35 to 1.49 to 2.00. However, Southern Xinjiang remained the same from 0.82 to 0.80 to 0.81. From 1.28 to 1.08 to 1.14, Eastern Xinjiang showed a slight decline.

**Table 2:** Ecological efficiency and deconstruction analysis of different regions from 2001 to 2015.

Area	Eco-efficiency	Ranking	Resource efficiency	Ranking	Environmental efficiency	Ranking
Eastern Xinjiang	1.1674	2	0.4997	3	1.0294	2
Southern Xinjiang	0.8090	3	0.5732	2	0.6676	3
Northern Xinjiang	1.6128	1	0.9015	1	1.1538	1

Northern Xinjiang, Eastern Xinjiang, and Southern Xinjiang have uneven development. Northern Xinjiang is larger than Eastern Xinjiang and Southern Xinjiang. Moreover, Northern Xinjiang and Eastern Xinjiang’s ecological efficiency are higher than 1.00, which is in the frontier of efficient production, while Southern Xinjiang is lower than 1.00, which is an ineffective area of ecological efficiency. It shows that Southern Xinjiang is backward in terms of economic development and sustainable development. Because 80 percent of the poor counties in Xinjiang are in Southern Xinjiang, Southern Xinjiang’s development level is backward. Although in Eastern Xinjiang, ecological efficiency is more than 1.00, the ecological efficiency shows a downward trend from 2001 to 2015, from the "Tenth Five-Year" " to the "Twelfth Five-Year" period. Because Hami City and Shanshan County in Eastern Xinjiang are both national resource-based prefectures, both are in the growth stage, and the development intensity is large, which belongs to high energy consumption and high pollution development.

### Prefectures level measurement and analysis

**Fig 6:**
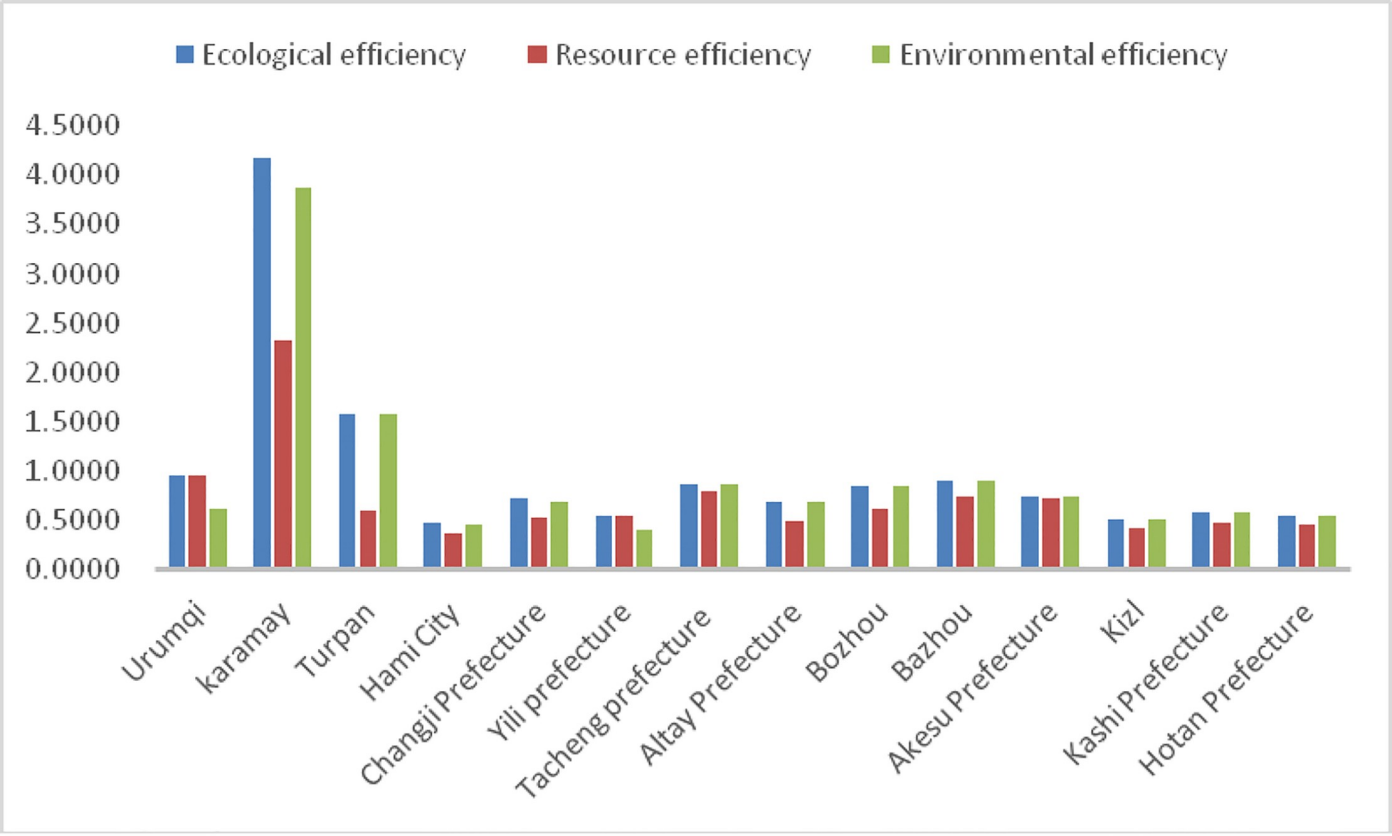
Comparison of ecological efficiency, resource efficiency, and environmental efficiency among 14 prefectures.

(1) Depending on the analysis of Fig 6, there was an imbalance in the ecological efficiency between prefectures. The mean ecological efficiency value for 14 prefectures was 1.02, where Karamay City had the highest ecological efficiency (4.19), which was 8.71-fold higher than the lowest ecological efficiency of Hami (0.48). However, the ecological efficiency values were low for the 14 prefectures. Karamay and Turpan City exhibited ecological efficiency values greater than 1.00, indicating that they had achieved the efficient production frontier. DMU’s ecological efficiency value intensity was subdivided into three categories [47, 48]. One of the components represented the efficient production frontier area where the ecological efficiency was higher than 1.00, including Karamay and Turpan City, which accounted for 14.3% of the total prefectures. The second component represented marginal non-efficient areas where the ecological efficiency ranged from 0.90 to 1.00, including Urumqi and Bazhou. The last component represented significantly weak areas where the ecological efficiency values were less than 0.90. Ten out of the 14 prefectures (71.4%) were separated into the inefficient area category and included Tacheng, Bozhou, Changji, and Aksu. The lowest is 0.48 in Hami City.

(2) As shown in Table 3, this paper decomposed the ecological efficiency into resource efficiency and environmental efficiency. Suppose higher than average represents high efficiency and lower than average as low efficiency. In that case, 14 prefectures consist of four types of development patterns [49]: The first development type was ‘low energy consumption and low emission’, an example of this type of region was Karamay City, where the resource and environmental efficiency were the highest in the 14 prefectures, and energy-saving and emission reductions were very efficient. The second development type was ‘high energy consumption and low emission’. Turpan is a typical example of this type, where resource efficiency was low, but environmental efficiency was superior. Though these areas achieved noteworthy reductions in emissions, energy conservation still requires improvement. The third development type exhibited low energy consumption and high emissions and included Urumqi, Tacheng, Bazhou, and Aksu, where resource efficiency was high, but environmental efficiency was low. These prefectures achieved remarkable success in energy conservation, but emission reduction should still be improved. The fourth development mode for prefectures takes high energy consumption and high emissions as an example. Many prefectures (57% of the total prefectures) fell into this type, including Bozhou, Kizl, Hami, the Changji Prefecture, and the Yili Prefecture, Altai Prefecture, Kashi Prefecture, and Hotan Prefecture. These areas had low resource and environmental efficiency. Energy conservation and emission reductions in these regions will require urgent improvement in the future.

**Table 3:** Development modes and characteristics of different prefectures.

Group	Characteristic	Mode	District
Group one	High resource efficiency	Low energy consumption and low emission mode	Karamay City
	High environmental efficiency		
Group two	Low resource efficiency	High energy consumption and low emission mode	Turpan City
	High environmental efficiency		
Group three	High resource efficiency	Low energy consumption and high emission mode	Urumqi, Tacheng Prefecture, *Bazhou, Akesu Prefecture
	Environmental inefficiency		
Group four	Low resource efficiency	High energy consumption and high emission model	Hami City, *Changji Prefecture, *Yili Prefecture, Altay Prefecture, *Bozhou, *Kizl, Kashi Prefecture, Hotan Prefecture
	Environmental inefficiency		

*Changji: Changji Hui Autonomous Prefecture

Yili: Kazak Autonomous Prefecture of Yili

*Bozhou: Boltala Mongol Autonomous Prefecture

*Bazhou: Bayinguoleng Mongolian Autonomous Prefecture

*Kizl: Kizlsukhz autonomous Prefecture.

The ecological efficiency was unbalanced among regions, out of sync. Due to Xinjiang’s developmental stage, wherein many of the prefectures are still in the primitive period of economic development. GDP growth was attributable to the development model of high-input and highpollution. Only a few prefectures exhibited high ecological efficiency and achieved sound and sustainable development, for example, the typical oil industrial city—Karamay. After more than 60 years of development, Karamay City has entered a mature industrial development stage, followed by sustainable development with low input and emissions. In contrast, Turpan City suffers from severe drought and water shortages. Due to the unique implementation of water-saving measures, sewage discharge in Turpan City was the lowest among all other Xinjiang regions, comprising a total of 0.38 tons/10,000 yuan of industrial GDP.

The ecological efficiency of Turpan City remained at the efficient production frontier. Urumqi and Bazhou are mainly based on industry, whose ecological efficiency is close to 1.00. In 2015, their GDP ranked the first and the third in Xinjiang, respectively. Their energy consumption is low, but their pollutant emission is high, which affects their ecological efficiency. However, most prefectures exhibited high energy consumption and high pollution. Thus, considerable scientific and technological research attention besides investment in environmental protection should be given to Xinjiang to achieve increased energy conservation and emission reductions, thereby narrowing the regional gap in ecological efficiency and achieving overall sustainable development.

### Dynamic ecological efficiency measurement and analysis

To better clarify the ecological efficiency change trend in Xinjiang, this paper used the DEA- Malmquist index model to calculate the ecological efficiency variations in 14 prefectures from 2001 to 2015. The Malmquist index included comprehensive technical efficiency (EC), technical progress (TC), pure technical efficiency (PE), scale efficiency (SE), and total factor productivity (TFP).

#### Analysis of time variation trend

As shown in Table 4:

(1) From 2001 to 2015, the mean change of total factor productivity(TFP) in Xinjiang was 0.939, showing a downward trend, with an average annual decline rate of 6.0%. Only in 2014–2015, TFP >1.000, was 1.021, showing an upward trend, with an increased rate of 2.1%. However, in other years, it showed a downward trend. In 2010–2011, the decrease rate was 21.3%, and in 2003–2004, the decrease rate was 14.5%. It is also the principal factor leading to the downward trend of TFP in Xinjiang.

(2) Depending on the analysis of changes in TFP decomposition indexes, the average technical progress index (TC) is 0.925 and showed a downward trend, with an average annual decline rate of 7.5%. However, the average comprehensive technical efficiency (EC) was 1.015, showing an average growth trend and an average annual growth rate of 1.5%. Except for a slight decline in a few years, the rest showed an annual growth trend, with the growth rate ranging from 0.3% to 10.9%. The comprehensive technical efficiency (EC) fell into pure technical efficiency (PE) and scale efficiency (SE) for analysis, the former increases by 1.5%, while the latter remains stable at 1.0. We can see that only the technical progress index (TC) shows a downward trend and keeps pace with the TFP index, which is the primary influence and constraint factor of TFP. In contrast, the technical efficiency index plays a promoting role.

(3) From the “Tenth Five-Year Period” to the “Eleventh Five-Year Period”, to the “Twelfth Five-Year Period”, from 0.927 to 0.958 to 0.934, total factor production(TFP) took the lead in rising and then falling. Overall there is a slight growth. The comprehensive technical efficiency (EC) and pure technical efficiency (PE) are higher than 1.000, showing an overall growth trend, the scale efficiency (SE) remains unchanged at 1.000, and the technical progress index(TC) declines first and then rises, showing a slight upward trend.

**Table 4:** Malmquist index of ecological efficiency in Xinjiang from 2001 to 2015.

Year	EC	TC	PE	SE	TFP
2001–2002	0.980	0.946	0.980	1.000	0.928
2002–2003	1.013	0.952	1.013	1.000	0.965
2003–2004	0.997	0.858	0.997	1.000	0.855
2004–2005	1.004	0.955	1.004	1.000	0.959
2005–2006	1.036	0.928	1.036	1.000	0.961
2006–2007	1.063	0.928	1.063	1.000	0.986
2007–2008	1.005	0.962	1.005	1.000	0.967
2008–2009	1.007	0.950	1.007	1.000	0.956
2009–2010	1.109	0.829	1.109	1.000	0.920
2010–2011	0.944	0.833	0.944	1.000	0.787
2011–2012	0.988	0.927	0.988	1.000	0.916
2012–2013	1.034	0.934	1.034	1.000	0.966
2013–2014	1.005	0.974	1.005	1.000	0.979
2014–2015	1.034	0.987	1.034	1.000	1.021
Mean	1.015	0.925	1.015	1.000	0.939
Tenth“Five-Year Plan", 2001–2005	0.999	0.928	0.999	1.000	0.927
Eleventh“Five-Year Plan", 2006–2010	1.044	0.919	1.044	1.000	0.958
Twelfth“Five-Year Plan", 2011–2015	1.001	0.931	1.001	1.000	0.934

#### Spatial distribution analysis

To further analyze the composition and causes of the Malmquist index changes in Xinjiang, we decomposed and analyzed the Malmquist indexes of 14 prefectures in Xinjiang, as shown in Table 5.

**Table 5:** The Malmquist index of eco- efficiency in 14 prefectures of Xinjiang.

district	EC	TC	PE	SE	TFP
Urumqi	0.993	0.952	0.993	1.000	0.946
Karamay City	1.000	0.945	1.000	1.000	0.945
Turpan City	1.035	0.927	1.035	1.000	0.959
Hami City	1.016	0.944	1.016	1.000	0.959
Changji Prefecture	1.043	0.947	1.043	1.000	0.988
Yili Prefecture	1.024	0.928	1.024	1.000	0.95
Tacheng Prefecture	1.012	0.942	1.012	1.000	0.954
Altay Prefecture	1.013	0.935	1.013	1.000	0.947
Bozhou	1.014	0.953	1.014	1.000	0.966
Bazhou	1.007	0.960	1.007	1.000	0.967
Aksu Prefecture	1.036	0.885	1.036	1.000	0.917
Kizl	1.000	0.820	1.000	1.000	0.82
Kashi Prefecture	1.012	0.914	1.012	1.000	0.925
Hotan Prefecture	1.008	0.902	1.008	1.000	0.909
Mean	1.015	0.925	1.015	1.000	0.940

As shown in Table 5:

(1) From 2001 to 2015, the mean change of total factor productivity (TFP) of all prefectures was 0.940, showing an average downward trend with an average decline range of 6.0%. TFP of all prefectures was less than 1.000, showing a downward trend. Among them, Kizl had the most extensive decline range of 18%, contributing to the most to the decrease of the TFP index in the entire region.

(2) Depending on the analysis of TFP decomposition indexes changes, the average technical progress index (TC) was 0.925, showing a downward trend on average, with an average annual decline rate of 7.5%. The TC index of various prefectures was less than 1.000, showing a downward trend, especially in Kizl, with an enormous decline rate of 18%. Moreover, the average comprehensive technical efficiency (EC) was 1.015, with annual growth of 1.5%. Besides, the EC was slightly decreased in Urumqi (decreased 0.7%), the rest of the prefectures were all on the rise. The comprehensive technical efficiency (EC) fell into pure technical efficiency (PE) and scale efficiency (SE) for analysis. The former increased by 1.5%, the latter held steady at 1.000. We can see that only the technical progress index (TC) showed a downward trend and kept pace with the TFP index, which was the primary influence and constraint factor of TFP. In contrast, the technical efficiency index played a promoting role.

Depending on the annual analysis results of the Malmquist index of 14 prefectures in Xinjiang, we further analyzed the causes of the Malmquist index change in Xinjiang.

As shown in Table 6:

(1) Although the TFP index of various prefectures was less than 1 for 15 years, and showing a downward trend. However, the annual analysis shows that there are still a few prefectures with a TFP index higher than 1 and showing a growing trend every year. In terms of frequency, the Turpan City and Changji Prefecture appeared six times, Hotan Prefecture appeared five times, Aksu Prefecture and Kashi Prefecture appeared four times, Urumqi and Karamay City appeared three times, Hami City and Tacheng Prefecture appeared two times, and the others appeared one time. It can be seen that the development positioning of each city in different years is different, so the development trend is also different. Although some prefectures have low ecological efficiency and are located in prominent inefficient areas, such as Changji Prefecture, Aksu Prefecture, Kashi Prefecture, Hotan, and Hami City, it does not affect their outstanding performance in some years, and total factor productivity shows an increasing trend. On the contrary, Karamay City and Turpan City, which are at the efficient production frontier, do not have outstanding performance. It is only in individual years that the TFP index is higher than 1. The above indicates that the development of different prefectures in Xinjiang is unstable, out of step, and short of sustainable.

(2) From the technical efficiency index and technical progress analysis, the principal factor leading to the decline of the TFP index in Xinjiang is the technological progress index. A low technical level causes the principal factors affecting the decline of total factor productivity for 15 consecutive years. Four years of the decline in TFP was the result of both technical efficiency and technical degradation. It indicates that the low technical level is the primary and critical factor restricting ecological efficiency in Xinjiang.

**Table 6:** Comprehensive statistical table of the Malmquist index in Xinjiang from 2001 to 2015.

Year	Prefectures of EC > 1	Prefectures of TC > 1	Prefectures of TFP> 1	TFP falling factors	Prefectures of “regressive range” > 15%
2001–2002	5	④⑤⑥⑧⑩⑾	④⑤⑥⑩	TC、EC	⑿
2002–2003	8	①②	③⑤⒁	TC	⒀
2003–2004	9	—	—	TC、EC	③⑧⑾⑿⒀⒁
2004–2005	9	⒀	⑤⒁	TC	—
2005–2006	12	—	⑦⒁	TC	—
2006–2007	13	—	③④⑾⒀	TC	⑦
2007–2008	11	①③⒀	③⑨⑾⒀	TC	⑿⒁
2008–2009	11	①③	③⑦⒁	TC	⑾⑿
2009–2010	10	③	③	TC	①②⑿
2010–2011	6	⑨	⑦⑨	TC、EC	②③④⑥⑧⑾⑿⒀⒁
2011–2012	11	①②	②	TC、EC	③⑦⑿⒁
2012–2013	12	—	⑤⒀⒁	TC	—
2013–2014	12	①②	②⑤⑾	TC	—
2014–2015	11	②③⒀⒁	②③⑤⑧⑾⒀	TC	—

①Urumqi ②Karamay City ③Turpan City ④Hami City ⑤Changji Prefecture ⑥Yili Prefecture ⑦Tacheng Prefecture ⑧Altay Prefecture ⑨Bozhou ⑩Bazhou ⑾Akesu Prefecture ⑿Kizl ⒀Kashi Prefecture ⒁Hotan Prefecture.

In terms of the number of prefectures where EC >1, most prefectures’ technical efficiency shows an increasing trend each year, which indicates that Xinjiang is well developed in the application and promotion of technology, and the annual growth trend is relatively stable. On the contrary, the number of prefectures where TC > 1 is tiny each year. In most years, only 1–2 prefectures showed an increase in the technological progress index. In some years, there is not one city with TC>1. It can be seen that Xinjiang is very weak in technology introduction and research and development, which has incredibly restricted the sustainable development of the economy, society, and environment in Xinjiang.

Regarding the occurrence frequency of prefectures where TC >1 each year, except for Tacheng Prefecture and Kizl, there were five occurrences in Urumqi, four occurrences in Karamay City and Turpan City, three occurrences in Kashi City, and one occurrence in the other eight prefectures. It can be seen that the technical level of Urumqi, Karamay City, and Turpan City, which is developed at the frontier and marginal frontier of ecological efficiency, is relatively high. In the introduction of technology, research and development are given more attention, but still insufficient. There is room for improvement. As far as Kizl and Tacheng Prefecture are concerned. It is very backward in terms of technology introduction and research and development.

(3) Through the analysis of prefectures with large regression (> 15%), there were nine years in which significant decline occurred. Among them, Kizl appeared six times, accounting for 66.7%; Turpan City, Aksu Prefecture, Kashi Prefecture, and Hotan Prefecture appeared three times, accounting for 33.3%; the rest appeared less than two times; Changji, Bozhou, and Bazhou appeared 0 times. Eleven prefectures showed a significant decline, including Karamay City, Urumqi, and Turpan City, a district with high ecological efficiency. Indicating that the development of these prefectures is not stable, there are big ups and downs; however, the ecological efficiency of Kizl has been reduced almost every year, which has s affected the sustainable development process of Xinjiang and becomes a weakness of the local economy in Xinjiang. Total factor productivity in Changji, Bozhou, and Bazhou did not decline significantly, indicating that the development was stable. For Aksu, Kashi, Hotan, and Kizl in southern Xinjiang’s four prefectures, the ecological efficiency is low. Moreover, the development fluctuates wildly. In the future, improvement and perfection should be made in these two aspects.

(4) In 2003–2004 and 2010–2011, total factor productivity was low, which was 0.855 and 0.787, respectively, and the TFP index decreased by more than 15% in six cities and nine cities, respectively.

### Input-output redundancy analysis

In this paper, the input redundancy rate is obtained by dividing each input variable’s slack by the corresponding input index value in Xinjiang from 2001 to 2015. The calculation results are shown in Table 7.

**Table 7:** Redundancy rate of the input-output index of eco-efficiency in different prefectures of Xinjiang.

Area	Employment	Fixed asset investment	The total water	SO2	NOx	Waste water emissions	COD	NH_3-_N	GDP
Urumqi	-57.27%	-8.29%	-3.72%	-36.74%	-38.76%	-46.80%	-65.35%	-78.29%	0.00%
Hami city	-51.93%	-58.36%	-73.02%	-75.02%	-56.69%	-51.93%	-83.38%	-74.39%	0.00%
Changji Prefecture	-26.49%	-33.74%	-51.89%	-36.23%	-50.42%	-26.49%	-82.26%	-73.86%	0.00%
Yili Prefecture	-86.89%	-45.18%	-94.68%	-58.03%	-57.75%	-58.28%	-94.62%	-88.26%	0.00%
Tacheng Prefecture	-68.60%	-13.55%	-81.12%	-11.59%	-26.47%	-11.59%	-91.71%	-78.75%	0.00%
Altay Prefecture	-83.22%	-49.77%	-97.57%	-87.42%	-31.07%	-38.50%	-94.87%	-86.52%	0.00%
Bozhou	-74.30%	-35.34%	-89.69%	-13.73%	-33.63%	-13.73%	-91.14%	-71.19%	0.00%
Bazhou	-59.27%	-24.79%	-90.31%	-9.15%	-15.64%	-30.69%	-93.41%	-79.33%	0.00%
Akesu Prefecture	-85.80%	-26.95%	-97.15%	-24.00%	-27.70%	-35.75%	-88.81%	-84.62%	0.00%
Kizl	-92.50%	-56.07%	-96.43%	-48.36%	-55.10%	-54.00%	-95.64%	-91.51%	0.00%
Kashi Prefecture	-92.01%	-51.99%	-97.72%	-40.35%	-53.99%	-49.12%	-93.95%	-91.74%	0.00%
Hotan Prefecture	-94.76%	-53.35%	-98.05%	-44.32%	-54.65%	-44.32%	-91.01%	-88.84%	0.00%
Mean	-72.75%	-38.12%	-80.95%	-40.41%	-41.82%	-38.43%	-88.85%	-82.28%	0.00%

Note: The table lists 12 prefectures with ineffective ecological efficiency.

**Fig 7:**
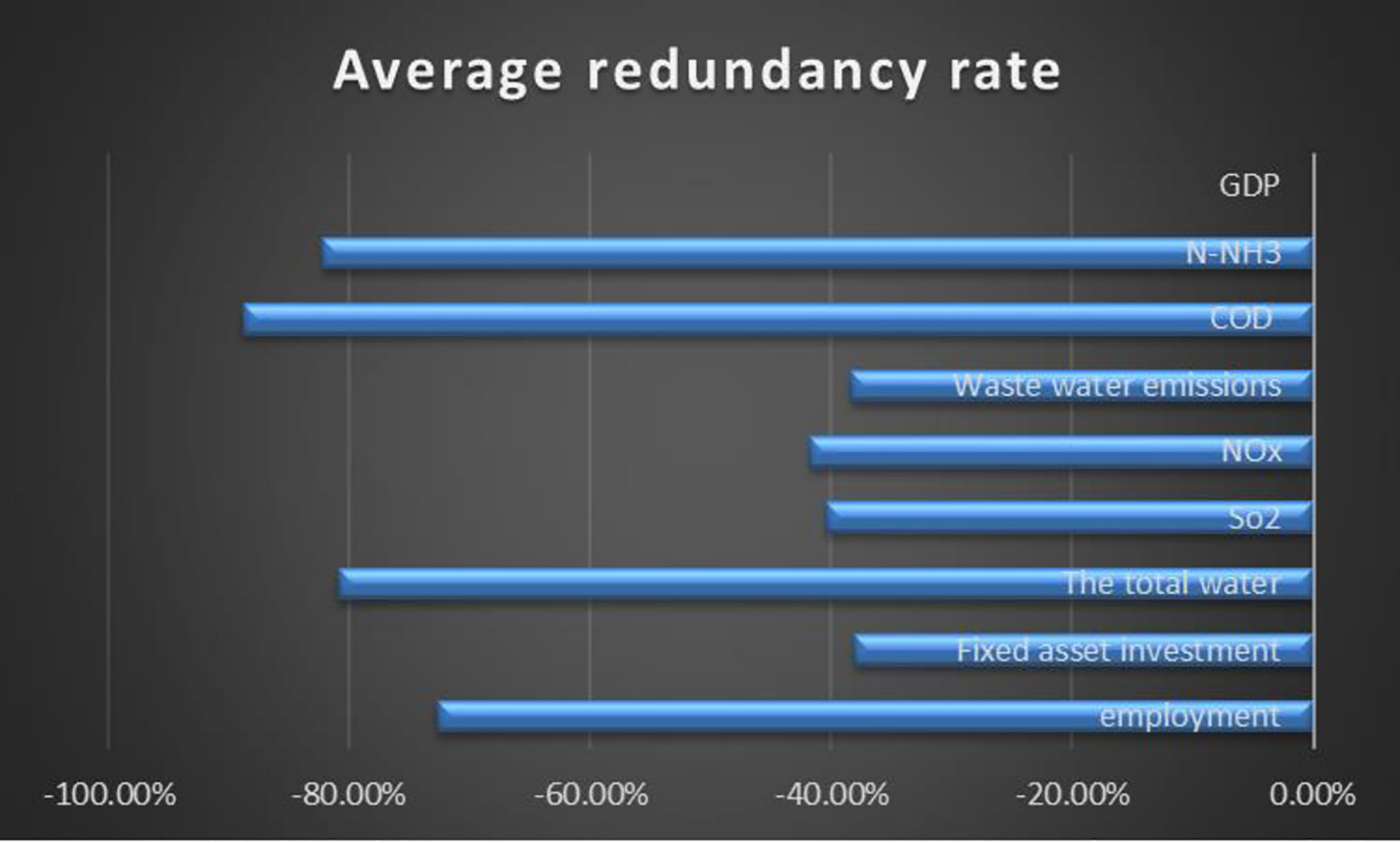
Comparison of redundancy rates of input and output index.

Conclusions can be drawn from Table 7, Fig 7:

(1) The redundancy rate of each region’s output index is zero, and there is the redundancy of input factors. It shows that insufficient output is not the cause of ecological efficiency loss, but excessive resource consumption and excessive emission of environmental pollutants are the leading causes of low ecological efficiency.

(2) From the mean redundancy rate, the principal influencing factors for the loss of ecological efficiency are: resource consumption input index includes water resource input and labor force input, and the environmental pollution emission index includes COD and NH_3_-N emission. We can observe that the total amount of water and drainage in all regions is excessive problems. Water conservation in arid areas is a key problem. How to increase the distribution and utilization of water resources is urgent in the next step. Excessive labor force and low labor productivity are also the principal reasons for the low ecological efficiency. In the next step, we should reduce redundant personnel, improve labor productivity, and transform the industrial structure from labor-intensive to technology-intensive.

(3) From the perspective of different regions, ecological efficiency loss’s principal influencing factors vary in different regions.

The redundancy rate of the eight input indexes all exceeded the average value in the Southern Xinjiang three prefectures (except Aksu) and Yili Prefecture, indicating that the development of these regions is still in the initial accumulation and development stage with high labor intensity, high input, high energy consumption, and high pollution, and the production efficiency is extremely low.

Except for nitrogen oxide and waste-water discharge, all the other six indexes in Altay Prefecture exceed the standards, among which sulfur dioxide exceeds the standards possibly related to mineral development and smelting. In Aksu Prefecture, Bozhou, Tacheng Prefecture, Bazhou, four primary indicators: Labor, water resources input, COD emissions, NH_3_-N emissions exceed the standard, consistent with the average redundancy rate. They are all problems of excessive water use and drainage, and surplus labor.

In Hami City and Changji Prefecture, sulfur dioxide and nitrogen oxide emissions exceeded the limit. It is related to the large number of industrial enterprises polluting the air and the large pollutant discharge load in these two regions.

### Factors influencing regional ecological efficiency

There are few current theoretical and empirical studies regarding the factors that affect ecological efficiency. This paper selected six indices for empirical analysis based on previous studies [25, 50]:

(1) Economic development level that was measured by per capita GDP; (2) The industrial structure that was estimated by the proportion of tertiary industry; (3) Utilization of foreign capital that was calculated by the proportion of foreign investment; (4) Urbanization rate that was inferred by the ratio of urban population to total population; (5) Average education level that was computed by the number of primaries, secondary, and university students in the region multiplied by their time of the study; and (6) Degree of marketization that was measured by the ratio of non-public officials to employees. Considering the above six indicators as independent variables and the value of ecological efficiency as the dependent variable, we carried out the Tobit regression analysis using Stata software.

As shown in Table 8, ecological efficiency was positively correlated with the proportion of foreign investment, the proportion of the urban population, and the average education level within prefectures (P < 0.05). However, ecological efficiency was negatively correlated with non-public officials’ proportion within a prefecture (P < 0.001). These results suggest that the utilization of foreign capital, urbanization rate, and education level played a considerable role in promoting a prefecture’s ecological efficiency. However, the marketization appeared to inhibit ecological efficiency. Based on the above results and considering Xinjiang’s practical realities, the following methods are recommended to improve the ecological efficiency of Xinjiang and ensure the coordinated and sustainable development of the environment, economy, and society in the future of the area. First, we should strengthen urbanization, especially the building of small prefectures and towns, to accelerate the implementation of the general process of well-off in line with the 18th and 19th CPC National’s urban development policies and the newly released rural revitalization plans. Second, we should enhance scientific and technological research. Besides, we should increase the application and transformation efficiency of scientific and technological achievements. Although progress has been evident in constructing a scientific and technological talent pool, the application of research and development is still in the exploratory and initial developmental stages. Third, we should adjust the environmental protection policy and improve the efficiency of governance. Fourth, we should strengthen the management of non-public enterprises, and we should not sacrifice environmental benefits for the sake of GDP. Fifth, we should encourage a foreign investment that is beneficial to the sustainable development of Xinjiang.

**Table 8:** Tobit regression analysis of factors influencing eco-efficiency.

Explanatory variable	Coefficient	Standard deviation	Z statistic	P -value	Significance
Constant term	5.305	0.182	-29.058	2e-16	***
GDP per capita	0.001	0.006	-0.106	0.915	
The proportion of tertiary industry	0.143	0.101	1.422	0.155	
The proportion of foreign investment	0.258	0.058	4.476	7.60e-06	***
The proportion of urban population	0.022	0.003	7.518	5.55e-14	***
Average education level	0.467	0.193	2.427	0.015	*
The proportion of Non-public officers	1.182	0.194	-6.082	1.19e-09	***

***, **, * represent statistical significance at the 0.001, 0.01, and 0.05 levels, respectively.

## Discussions

**The overall ecological efficiency of Xinjiang is low**, but from 2001 to 2015, the ecological efficiency of Xinjiang showed a W-shaped fluctuation trend. Depending on the measurement analysis, the ecological efficiency of Xinjiang ranks the 31st in China in 2015, only 0.5622, not reaching the efficient production frontier, which is nearly twice lower than the average and 4.32 times different from the first place Beijing. The resource efficiency ranks 30th, and the environmental efficiency ranks 22nd in China. It shows that the principal reasons for the low ecological efficiency in Xinjiang are low resource efficiency, unreasonable resource allocation, large resource consumption, and high energy consumption, which impact the sustainable development of Xinjiang. From 2001 to 2015, the ecological efficiency of Xinjiang fluctuated and finally increased by 5.7%. From 0.9881 during the Tenth Five-Year Plan period to 1.0638 during the Eleventh Five-Year Plan period and 1.0643 during the Twelfth Five-Year Plan Period, there are small fluctuations, but the situation is rising in stages. In particular, from the beginning of the eleventh Five-Year Plan in 2006 to the end of the twelfth Five-Year Plan in 2015, except for a slight decline in 2009, the ecological efficiency has been stable at more than 1, keeping in the efficient production frontier. It shows that since 2001, through a series of policy measures and the implementation of the three five-year plans, Xinjiang’s ecological efficiency has been improved and enhanced in stages, which is due to a sharp growth of environmental efficiency, increasing by 2.35 times, growth of 135.7%, from 0.4771 at the beginning of the tenth Five-Year Plan in 2001 to 1.1259 at the end of the twelfth Five-Year Plan in 2015, and achieved a leap from low environmental efficiency to high environmental efficiency. It shows that from the tenth five-year Plan to the twelfth Five-Year Plan, through 15 consecutive years of prevention and control of environmental pollution, Xinjiang has achieved remarkable environmental governance results andimproved its environmental efficiency.

**Development among regions is unbalanced, out of sync, lacks sustainability**, and ecological efficiency level in all regions is low. Xinjiang consists of three regions. The ecological efficiency of Northern Xinjiang is greater than that of Eastern and Southern Xinjiang, and Southern Xinjiang is the lowest among them, not reaching 1. In the comparison of ecological efficiency among 14 prefectures, the difference between the highest and the lowest was 8.71 times, and only Karamay City and Turpan City had the ecological efficiency value higher than 1, which reached the efficient production frontier, indicating that the level of ecological efficiency was low in most prefectures. Furthermore, the difference was enormous. We compared the variation trend of ecological efficiency in different prefectures from 2001 to 2015, and there are equally significant differences among different prefectures. The annual variation trend of ecological efficiency in different prefectures is unbalanced and out of sync. There are four development and variation trends in 14 prefectures, and there are also frequent annual fluctuations. It shows that the development between regions does not follow the unified plan, and the development between regions is independent, and the plan is adjusted, which is very disorderly.

### The common problem in arid areas is the shortage of water resources and the fragile ecological environment caused by water resources shortage

Water resources are the restricting factor for the development of arid areas. The key to improving ecological efficiency is to save water resources and improve the efficiency of the comprehensive utilization of water resources. Turpan city is a city with extreme drought and water shortage. Due to the unique implementation of water-saving and water recycling measures, the discharge of sewage is only 0.38 tons/10,000 yuan of industrial GDP, which is the lowest in Xinjiang. Thus, the ecological efficiency level is high, and the efficient production frontier is reached. From the analysis of the input-output redundancy rate, we can see that the principal factors causing ecological efficiency loss are water resources input, labor input, COD emission, and ammonia nitrogen emission. It shows that the total amount of water and the total amount of drainage in the entire region is all excessive, so it is urgent to improve the distribution and utilization of water resources in the next step to save energy and reduce emissions.

### As a resource-rich province, Xinjiang is full of coal, oil, natural gas, and other mineral resources, ranking second in China [31]

Among the 14 prefectures in Xinjiang, eight are listed as resource-based regions by the Chinese Government [32], accounting for 60 percent of the prefectures in Xinjiang. Resource-based prefectures impact ecological efficiency [51], leading to low ecological efficiency and traps of a low level of ecological efficiency [52]. Resulting from this factor, Xinjiang ranks at the bottom in China in terms of ecological efficiency. Among them, the ecological efficiency level of Hami City is the lowest in Xinjiang, only 0.48, which is linked to the fact that Hami City is a coal mine development city. Mineral exploitation will aggravate the damage to the ecological environment. For Xinjiang, an arid and ecologically fragile region, resource exploitation has a significant effect on sustainable development. Thus, it is essential to pay equal attention to both development and protection.

### Uncertainty analysis. The difference of analysis models, data quantity, and the input-output index will produce a definite influence on the research results and make the research conclusions produce deviation

For example: In this study, the classification of the development patterns of 14 prefectures is different from the previous studies [34]. We analyzed the causes, one is the use of methods, such as the research of Zhigang Gao et al. used the traditional data envelopment methods (DEA), but the super-efficiency DEA model is used in this study. The article [35] has made a comparison. The traditional DEA model can only be calculated up to 1. Once you get to 1, it becomes qualitative. However, the super-efficiency DEA model can calculate the accurate data after 1. For example, we use the super-efficiency DEA model to calculate the ecological efficiency of Karamay as 4.18, while the traditional DEA model can only be calculated as 1.This leads to errors in calculating averages. Second, the data used are different. We used the average data of the 15 years from 2001 to 2015 to calculate the ecological efficiency value of each prefecture, while Zhigang Gao et al. only used the data of two sections in 2005 and 2010 to calculate, which can only represent the data of a certain year. Third, different input index parameters are used. Zhigang Gao et al. chose three resource input indexes of land, energy, and water, while in this study, five input indexes of resources, electricity, water, capital, and labor are selected. Thus, the error of the results of this study is small, and the data is more scientific than others.

### Although many scholars have done much work on the factors affecting regional ecological efficiency, a relatively complete research system has not been formed

This study summarized the influencing factors of ecological efficiency into three aspects. First are internal factors, including resource input indicators, environmental indicators, and economic indicators. This study shows that water resources input, labor input, COD, and ammonia nitrogen emissions are significant constraints to the ecological efficiency of Xinjiang. Second, are external factors. This study’s conclusion shows that foreign capital utilization, urbanization rate, average education level, and ecological efficiency are significantly positively correlated, which is in line with the research results of Hao Chen et al. [53] and Jiefang Xu et al. [54]. However, the degree of marketization is negatively correlated with ecological efficiency. The third is the impact of the Malmquist index. This factor affected by local conditions and different regions has different conclusions. This study shows that the technological progress index impacts and restricts Xinjiang’s ecological efficiency, while the comprehensive technological efficiency index, pure technological efficiency index, and scale efficiency index have a promoting effect on the ecological efficiency of Xinjiang.

### Social stability and order are key factors affecting sustainable development

The study shows that total factor productivity (TFP) was very low in 2003–2004 and 2010–2011, with 0.855 and 0.787 respectively, and the TFP index declined by more than 15% in 6 and 9 prefectures. By analyzing the factors, 2003–2004 was the year of the SARS outbreak, and the country mobilized all its resources to fight against SARS. The economic construction was not normal, so the ecological efficiency level dropped dramatically. The year 2010–2011 was also an extraordinary year for Xinjiang. As most of our efforts focused on maintaining social stability, economic construction cannot be kept normal. It indicates that social emergencies will temporarily disrupt the rhythm of social and economic development and cause significant fluctuations and declines in social development. So we can figure out that a stable social order is a significant factor affecting the overall improvement of economic development and ecological efficiency.

### This study has guidance and reference function for studying the arid resource area

This study measures and analyzes ecological efficiency from the spatial perspective of the provincial level, regional level, and prefectural levels. From the "Tenth Five-Year Plan", "Eleventh Five-Year Plan", "Twelfth Five-Year Plan", three five-year plans demonstrate the changing trend of the ecological efficiency of Xinjiang. It has guidance and reference for the sustainable development of Xinjiang- a critical passage of the Silk Road Economic Belt and provides reference and reference for other arid resource-based regions’research work.

## Conclusions and suggestions

### Conclusions

Xinjiang has a low level of ecological efficiency, which has not reached the efficient production frontier. It is due to low resource efficiency. It shows that the resource allocation in Xinjiang is unreasonable, the resource consumption is enormous, and the energy consumption is high.

Development among regions is unbalanced, out of sync, lacks sustainability, and ecological efficiency in all regions is low. The ecological efficiency in Northern Xinjiang is higher than that in Eastern Xinjiang and Southern Xinjiang. The difference between the highest and lowest ecological efficiency was 8.71 times. From 2001 to 2015, the trend of ecological efficiency changes in different prefectures was unbalanced and out of sync.

From 2001 to 2015, the ecological efficiency of Xinjiang showed a W-shaped fluctuation rising trend and finally increased by 5.7%. It is due to the substantial improvement in environmental efficiency, from 0.4771 at the beginning of the tenth Five-Year Plan in 2001 to 1.1259 at the end of the twelfth Five-Year Plan in 2015, an increase of 2.35 times or growth of 135.7%, realizing a leap from low environmental efficiency to high environmental efficiency.

Water resources are restricting factors of the development of arid areas. The input-output redundancy analysis shows that excessive input of water resources, excessive COD, and ammonia nitrogen emissions are the principal restricting factors affecting ecological efficiency.

Depending on the classification of 14 prefectures in Xinjiang, most prefectures belong to the mode of high energy consumption and high emission. It is still the current development situation in most Xinjiang areas.

Through the analysis of the Malmquist index, we find that the technological progress index is the principal factor affecting and restricting ecological efficiency. The comprehensive technical efficiency index, pure technical efficiency index, and scale efficiency index play a unique role in promoting ecological efficiency.

From the analysis of external influencing factors, foreign capital utilization, urbanization rate, and average education level are positively correlated with ecological efficiency, while the degree of marketization is negatively correlated with ecological efficiency.

### Suggestions

Our suggestions are as follows:

We should strengthen unified planning, adjust the industrial structure, and allocate resources more rationally, strengthen the unified planning and deployment of Xinjiang, maintain the steady development of all prefectures, and promote the overall development momentum of the four prefectures in southern Xinjiang. Besides, we should adjust the industrial structure, optimize the primary industry, upgrade the secondary industry, expand the tertiary industry, build an industrial circular chain, improve the rationality of resource allocation and resource utilization, promote the advanced to drive the backward, give play to the advantages of each region, strengthen weak links, and ensure the synchronized and balanced development of all prefectures in Xinjiang.

We should further transform the pattern of economic development, promote economic transformation, and improve the quality of the economy; change the mode of production that is energy-intensive and highly polluting, accelerate the upgrading of the secondary industry, especially mineral development and processing enterprises, and build a modern economic system that meets the characteristics and high-quality requirements of Xinjiang. What’s more, we will promote energy conservation and consumption reduction by the law, properly control the increase in energy consumption, decrease energy intensity year by year, and keep it within the scope of constraint targets. Industrial prefectures will also follow the route of modern industrial development of energy conservation and emission reduction in Karamay City, while agriculture follows the road of energy conservation and water conservation in Turpan City.

We should implement the strategy of driving scientific and technological innovation, introduce new technologies, accelerate the transformation of scientific and technological achievements, establish a sound mechanism for talented people, and further improve the level of research, development, and dissemination of technologies; at the same time, the application of new technologies continues to be maintained and strengthened.

To solve the problem of high water consumption and displacement, we should implement the strictest water resources management system; the water resources of significant rivers and lakes should be developed and utilized in a scientific and orderly way; the healthy water ecology should be maintained, and the policy of fixed production and fixed land-based on the water should be implemented. Moreover, we will do excellent work reducing emissions, checking emissions at the source, and ensuring that ecological thresholds, environmental quality, and resource utilization are met.

We should strengthen urbanization and scientific and technological research and application; adjust environmental protection policies to ensure that they are more targeted and effective. Besides, we will strengthen the management of non-public enterprises; improve the quality of foreign investment introduction; set certain entry threshold, strictly check the threshold, select low energy consumption, low pollution, conducive to the sustainable development of Xinjiang high-quality enterprise projects, resolutely prevent "three high" enterprises and destructive capital projects into the region. Furthermore, we should streamline redundant personnel, improve labor productivity, transform the industrial structure from labor-intensive to technology-intensive.

## Supporting information

S1 File (ZIP)
