# Supplementary figures and images for: Sustainability assessment and improvement strategies research for typical arid and resource-developing regions
Source: PLoS One. 2021 May 4;16(5):e0251088. doi: 10.1371/journal.pone.0251088 (PMC8096093; doi:10.1371/journal.pone.0251088)

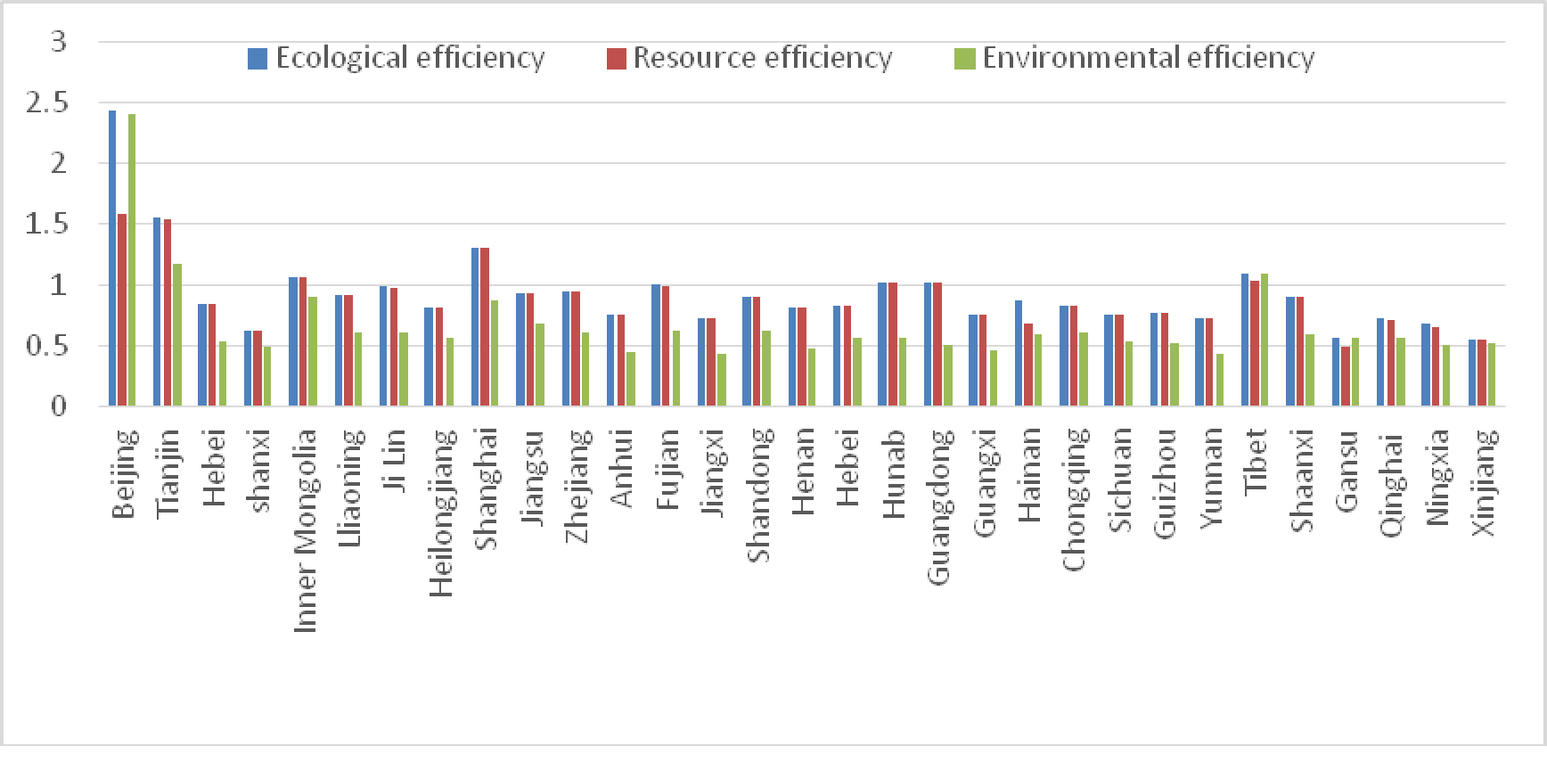

Supplement: S1 File — (ZIP) [file pone.0251088.s001.zip › Figures/Fig 1.tif]

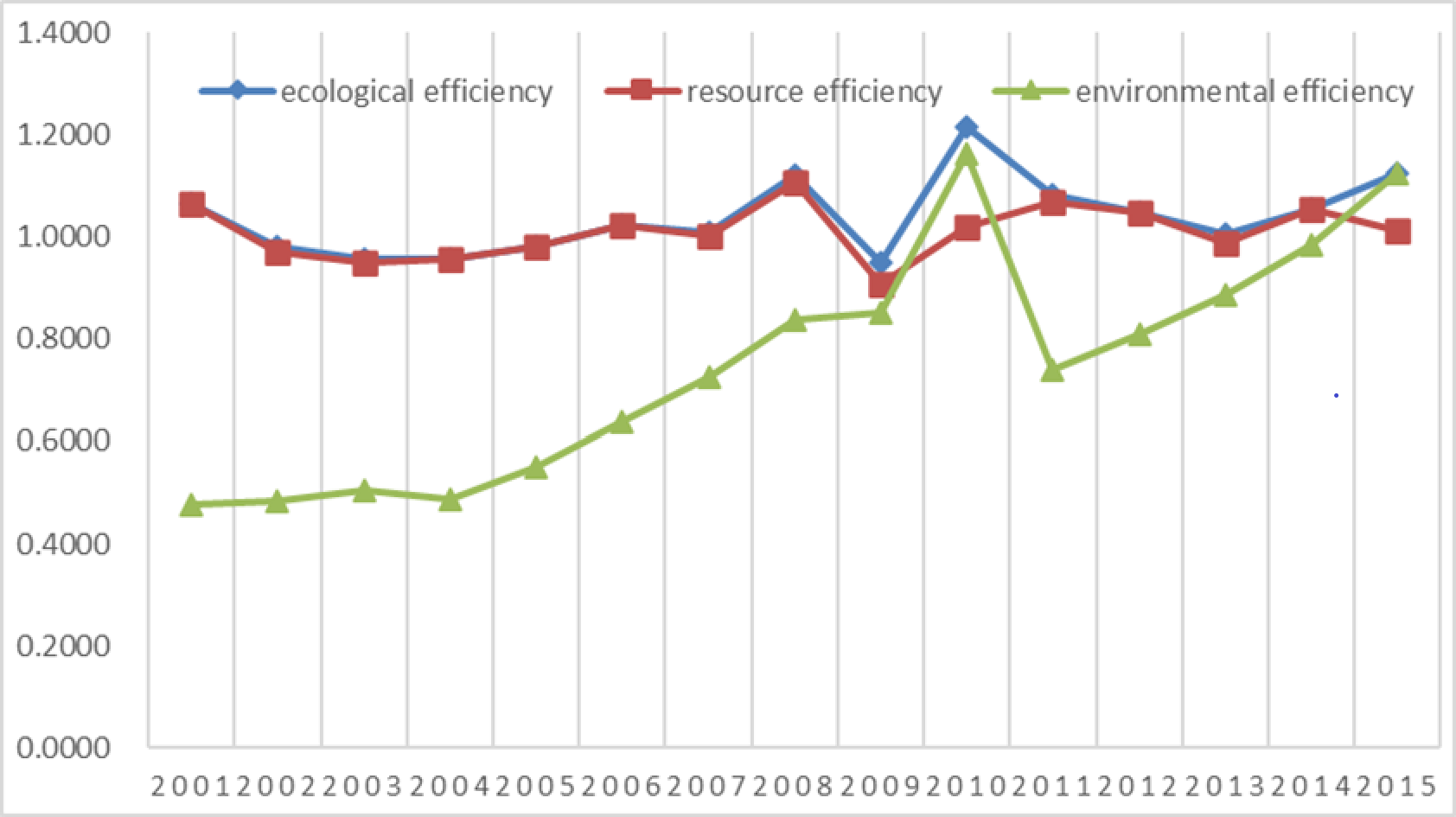

Supplement: S1 File — (ZIP) [file pone.0251088.s001.zip › Figures/Fig 2.tif]

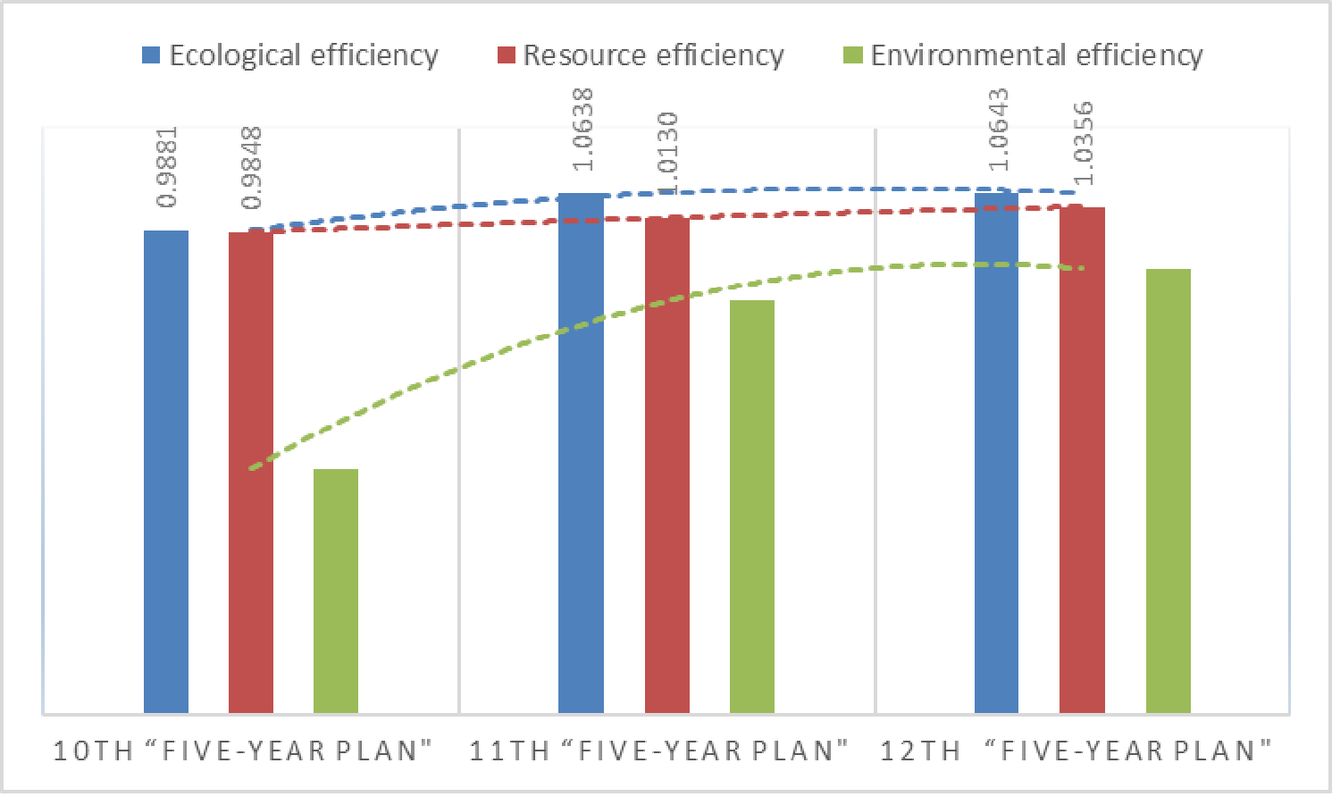

Supplement: S1 File — (ZIP) [file pone.0251088.s001.zip › Figures/Fig 3.tif]

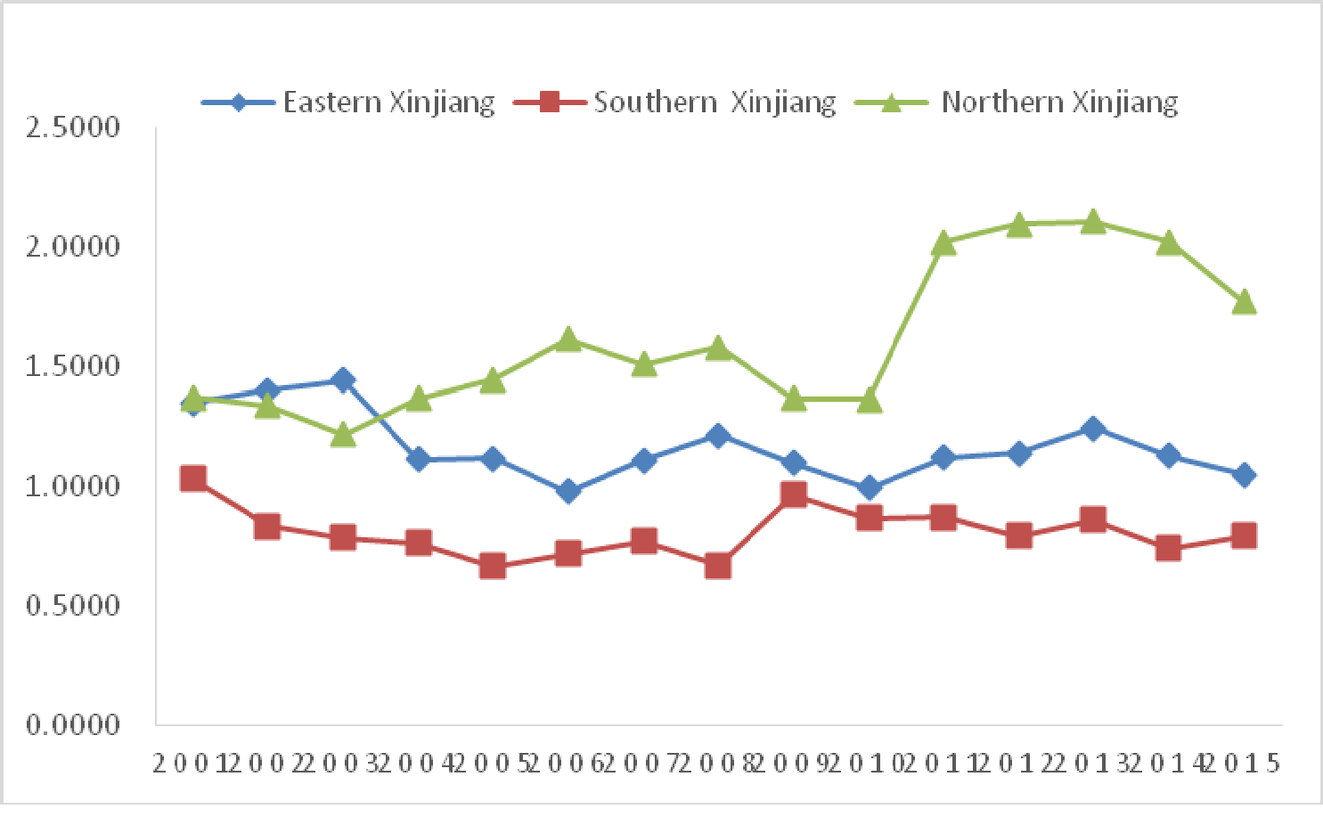

Supplement: S1 File — (ZIP) [file pone.0251088.s001.zip › Figures/Fig 4.tif]

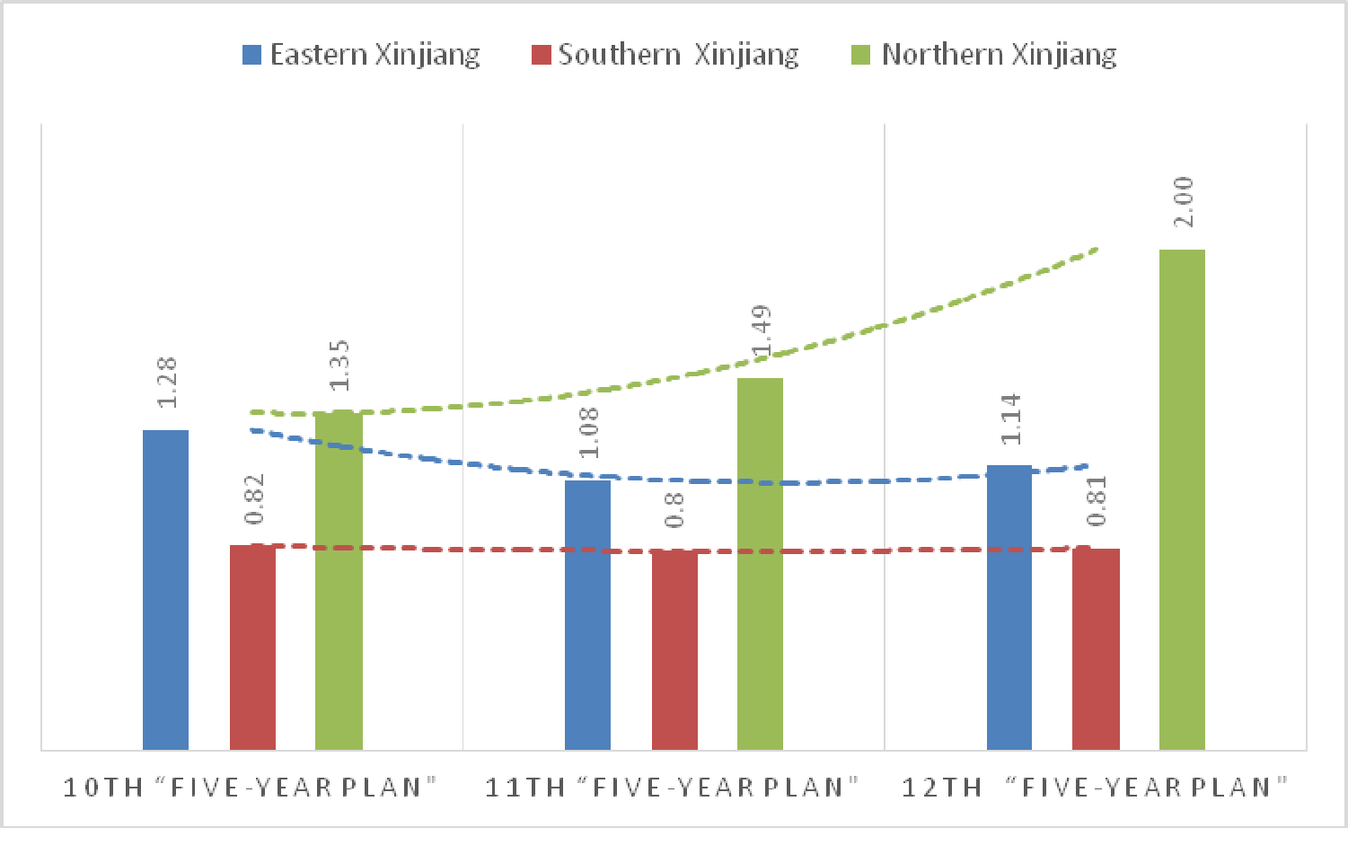

Supplement: S1 File — (ZIP) [file pone.0251088.s001.zip › Figures/Fig 5.tiff]

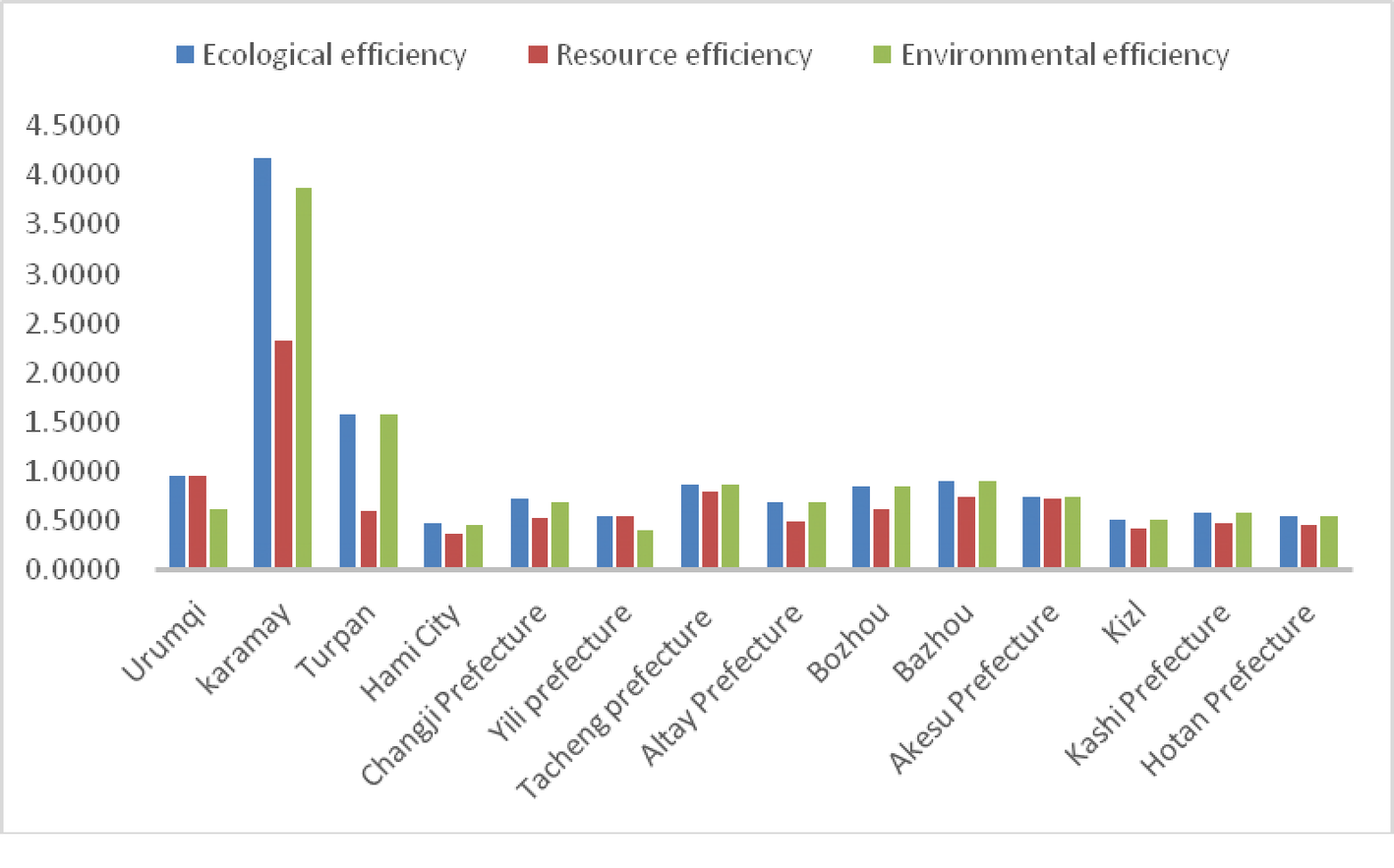

Supplement: S1 File — (ZIP) [file pone.0251088.s001.zip › Figures/Fig 6.tiff]

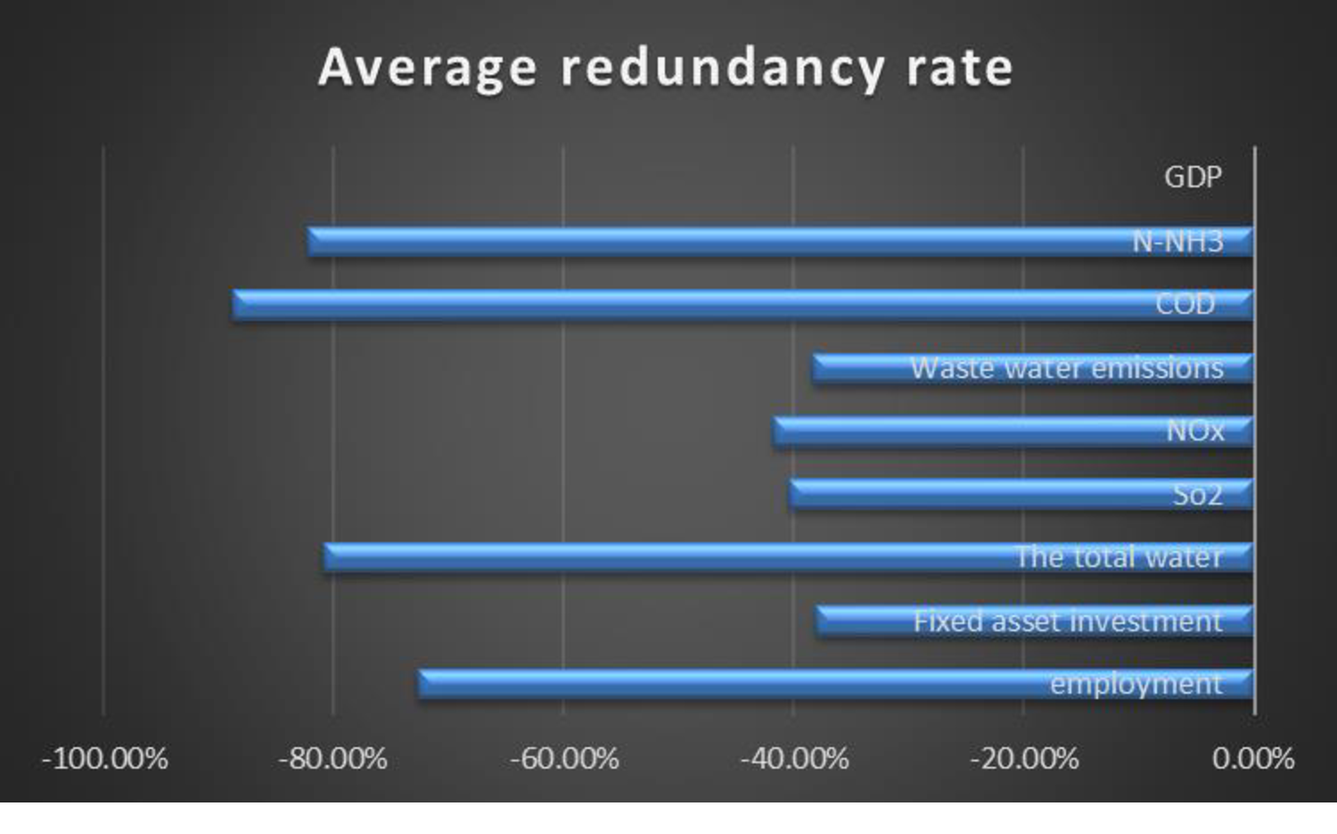

Supplement: S1 File — (ZIP) [file pone.0251088.s001.zip › Figures/Fig 7.tiff]
